# Entomopathogen-based biopesticides: insights into unraveling their potential in insect pest management

**DOI:** 10.3389/fmicb.2023.1208237

**Published:** 2023-07-26

**Authors:** Irsad, Mohammad Shahid, Ejazul Haq, Abdullah Mohamed, Parvez Qamar Rizvi, Elango Kolanthasamy

**Affiliations:** ^1^Department of Plant Protection, Faculty of Agricultural Sciences, Aligarh Muslim University, Aligarh, India; ^2^Plant-Microbe Interaction and Rhizosphere Biology, ICAR-NBAIM, Kushmaur, India; ^3^Research Centre, Future University in Egypt, New Cairo, Egypt; ^4^Kumaraguru Institute of Agriculture, Tamil Nadu Agricultural University (TNAU), Coimbatore, India

**Keywords:** biopesticides, potential entomopathogens, insect pest management, plant health, sustainability

## Abstract

Global food security is a critical challenge to fulfill the demands of an exponentially growing population. To date, growers rely on chemicals; the broad-spectrum application of synthetic molecules leads to environmental contamination, resistance development, residual toxicity, pest resurgence, and a detrimental effect on human health and cattle. Crop production needs to be improved considering environmental and human health concerns to ensure food security. Furthermore, economically important crops are prone to attack by insect pests, causing considerable yield losses. Microbes are an eco-friendly, versatile alternative, and a potential candidate for combatting destructive pests below the economic injury level and improving the plant's health and productivity. Several microbial pathogens, including parasites, predators, parasitoids, pollinators, and many beneficial microorganisms, possess toxic properties against target organisms but do not cause harm to the non-target organisms. Entomopathogens (ENMs) have great potential for pest suppression due to their remarkable properties. Bacteria are host-specific, but fungi have a broader host range and can be significantly affected by both soil-dwelling and terrestrial insect pests. Virulent pathogens cause mortality in target insect pests known as ENMs and can penetrate through natural openings, ingestions, and integuments to cause a possible effect on target insect pests. The objective of using ENMs is to sustain productivity, improve environmental health, reduce pesticides, and conserve natural resources. Moreover, research is ongoing to discover other possible aspects, especially exploring potential ENMs. Therefore, there is a need for identification, isolation, and bioformulation to overcome the existing issues. This study is mainly focused on the status of bio-formulations, pathogenicity, their mode of action, and the potential application of different types of microbial formulations for sustainable pest management.

## 1. Introduction

Insects require adequate nutritious food for survival and metabolism and search for their food through migration or distribution. They are considered successful creatures because of their wide distribution and tremendous climatic adaptability. Food scarcity impacts insect growth and reproduction and can even result in mortality (Zhang et al., 2019). Crops are prone to attack by various insect pests, and it has been recorded that insect pests have a significant impact on the nutritional and economic values of agricultural produce. There is an urgent need to overcome pest infestation through the potential application of crop protection measures.

Several insecticides are commercially available and extensively applied to suppress pest density, but these synthetic agricultural chemicals negatively impact agro-ecosystem, crop productivity (Shahid and Khan, 2017, 2018, 2022; Shahid et al., 2017, 2018, 2019, 2021a,b; Khan et al., 2020), human health, and diverse beneficial non-target organisms. Moreover, the repeated application of the same group of pesticides leads to resistance development and pest resurgence of the insect pest population (Abrol and Shankar, 2016; Islam, 2017). Several successful attempts to manage insect pests have been made to reduce pesticide exposure by conserving the environment and preventing pollution (Haase et al., 2015). As a result, an alternative and ecologically sound strategy to improve the nutritional quality and quantity of agricultural produce for long-term crop protection has been adopted extensively (Yadav et al., 2018; Kiran Kumar et al., 2019; Hesham et al., 2021). Due to the adverse effects of insecticides, biopesticides are needed because of their eco-friendly, safe, highly effective, and quickly decomposable properties. Biopesticides manufactured from microbiomes have a non-toxic approach to controlling the insect pest population. Many agents, such as viruses, fungi, and bacteria, are used to manage the insect pest population (Rastegari et al., 2020). More than 100 pathogenic bacteria species are being extensively used in managing the insect pest population, with the entomopathogenic bacteria *Bacillus thuringiensis* most widely used. Despite the fact that *B. thuringiensis* has been demonstrated to be insightful for its entomopathogenic actions.

Apart from bacteria, fungi have also been recognized as potential candidates and are successfully employed in pest management programs. The most common entomopathogenic fungi (EPFs) are *Beauveria, Metarhizium, Hirsutella, Verticillium, Lecanicillium*, and *Paecilomyces*.

Entomopathogenic fungi have a wide host range, toxicity, and ability to suppress chewing and sucking insect pests, and have gained a significant position as a biocontrol agent (Khan et al., 2012). Entomopathogenic microbiomes are generally more eco-friendly, more suitable, specific, and less expensive. The effect of ENMs has been tested on non-target organisms, and human health demonstrated satisfactory results (Yadav et al., 2018; Kumari et al., 2020). Their use is justified, and they can survive in the natural ecosystem. Soil microbes, including bacteria and fungi, also help in the decomposition of organic matter and the reutilization of dead plant materials; plants quickly absorb nutrients from decomposing organic matter (Schmeisser et al., 2007). Fungal biocontrol agents are far more specific and have a distinct mechanism of infection. They penetrate their hosts directly through the cuticle, in contrast to bacteria and viruses, which require ingestion to thrive (Mejía et al., 2008; St. Leger and Wang, 2010). Fungi cause damage to the host by producing spores that germinate on the host's surface and subsequently proliferate inside the host's body, resulting in infection (Brivio and Mastore, 2020). The infection rate is determined by the species of fungus and the number of infectious spores (Lu and Leger, 2016). The fungus continues to develop new spores on the deceased host body after infection and death of the target organism. These spores will disperse and persist on new hosts (Jaber and Ownley, 2018). Several researchers have highlighted the various insect-pathogenic fungal species as natural colonizers/endophytes of a diverse variety of economically valuable crops, such as maize (*Zea mays*), coffee (*Coffea arabica*), potato (*Solanum tuberosum*), cotton (*Gossypium* spp.), tomato (*Solanum lyocpersicum*), and chickpea (*Cicer arietinum*) (Arnold and Lewis, 2005; Qayyum et al., 2015).

**Figure 1:**
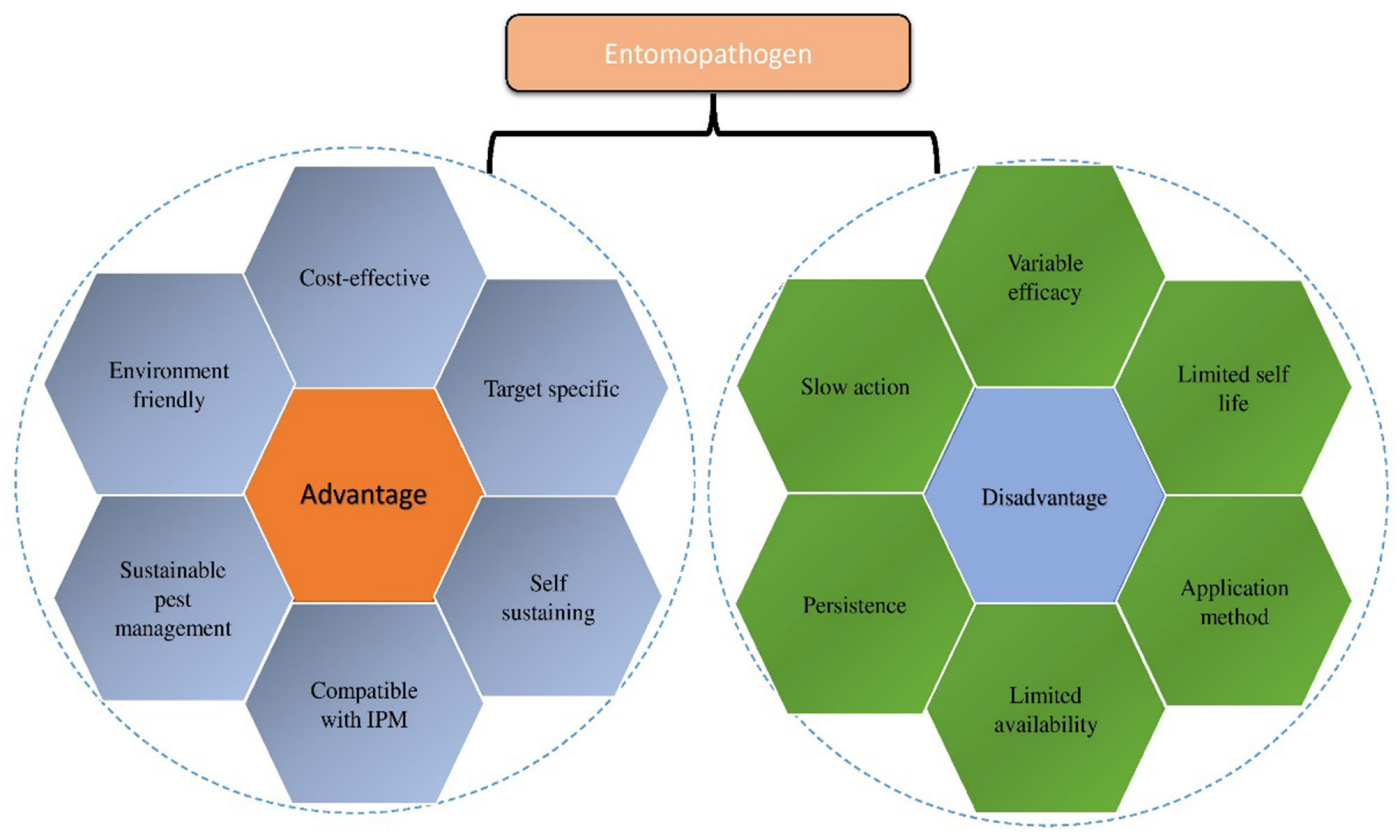
Graphical illustration of Entomopathogens (advantages and disadvantages).

Entomopathogenic fungi are a diverse and systematized heterogeneous category with varying biology. The majority of EPF are pathogenic to insects (Shah and Pell, 2003; Scholte et al., 2004; Vega et al., 2009; Dash et al., 2018), with a high degree of efficacy in infecting their host and acting as a regulator for reducing the population of detrimental insects (Ortiz-Urquiza and Keyhani, 2013; Vidal and Jaber, 2015; Lu and Leger, 2016). Several bacterial species belonging to the family Bacillaceae have been investigated and recorded as pathogenic to invertebrates, especially insects (Castagnola and Stock, 2014; Ruiu, 2015). The most studied and extensively used bacterial species is *B. thuringiensis*. In bacteria, some crystal toxins (Cry and Cyt) are produced during the sporulation phase, and other toxins are also produced and released during the vegetative stage of growth. It is made by the genetic modification of bacteria, fungi, algae, viruses, protozoans, and entomopathogenic nematodes. These are produced toxins and damage the integument and gut of the insect. This review highlights the potential application of microbial formulations to reduce or suppress the pest population and enhance sustainable crop production. It is an innovative and ecological tactic, significantly employed to tackle the insect pest population, with the environment, ecosystem, and human health concerns regarded as biopesticides (Fang et al., 2014). The degree of acceptability and adoption of entomopathogens is growing substantially due to the overall performance in laboratory and field conditions; thus, investigation into their biology, ecology, and mechanism of action is gaining more scientific attention (Dong et al., 2016). Several microbial biopesticides have been discovered, developed, and commercialized in recent decades as a result of several academic and corporate research efforts. Despite their enormous potential in biocontrol operations, the application of ENMs has been neglected due to a lack of awareness among growers. In this context, this review analyzes the current state of knowledge on ENMs utilization and mechanisms as biological agents for plant growth promotion and pest suppression, consequently addressing the prospects and limitations of their adoption as alternatives to synthetic pesticides (Figure 1).

## 2. Entomopathogenic bacteria

Most bacterial species belonging to the family Bacillaceae and their pathogenicity have been tested in invertebrates, especially insects (Ruiu, 2015). The most commercially used bacterial species are *B. thuringiensis*, a gram-positive and spore-forming bacterium. The sporulation process produces proteins with insecticidal properties in a parasporal crystal, comprising Cry and Cyt toxins, known as delta-endotoxin. Sometimes, cells release some compounds during vegetative development (De Maagd et al., 2001). Entomopathogenic bacteria are employed to suppress insect pests.

### 2.1. *Bacillus thuringiensis* subsp. kurstaki (*Btk*)

Government research institutes collaborate with business enterprises to ensure that *Btk* products are readily accessible in India. The IIOR has popularized manufacturing indigenous *Btk* isolates (DORBt-1 and DORBt-5) using solid-state fermenter technology for distribution and sale to suppliers. In addition, the NBAIR has industrialized liquid fermentation technology for the indigenous *Btk* isolates PDBCBT1 and NBAIIBTG4 (Ramanujam et al., 2014), which are also licensed for manufacture and sale. In India, numerous *Btk* formulations are used to combat bollworms, loopers, and other lepidopteran pests. In Andhra Pradesh (Kumara et al., 2016), Telangana (Vimala Devi and Vineela, 2015), and Punjab (Kumar and Kaur, 2017), for example, liquid *Btk* formulations have been effectively tested and used to manage *Helicoverpa armigera* and noctuid pests in pigeon pea. In sugarcane in Tamilnadu, commercial formulations (Delfin, Biobit, Dipel, and Halt) decreased the shoot borer, *Chilo infuscatellus*, below economic limits (Kesavan et al., 2003). *Btk* has proven to be effective against a variety of citrus pests. A study conducted in Andhra Pradesh, India (Rao et al., 2015) observed up to 100% mortality of the citrus leaf miner, *Phyllocnistis citrella*, for up to 10 days after spraying. The application of Dipel (1 kg/ha) resulted in a 90% decrease in the larvae population of the citrus butterfly, *Papilio demoleus*, in the fruits of sweet orange (Gopalakrishnan and Gangavisalakshy, 2005).

These and other studies showed that *Btk* is widely employed in various crops. Different control approaches, such as biological control agents, are compatible with these, although they are most successful when applied on the first and second instars of immature larvae. On the other hand, residues of Btk decay quickly in the sun and rain (Van Frankenhuyzen, 2009), necessitating reapplication, especially when insect numbers are high.

The Indian subcontinent has many Bt strains, some containing new assumed genes. Reyaz et al. (2017) discovered 68 Bt strains from the Kashmir Valley with four crystalline inclusion types. Mishra et al. (2017) identified 45 Bt strains from a new ecological niche in the Uttarakhand Himalayas, several of which (UKBt3, UKBt11, UkBt13, and UKBt18) exhibited promising approaches against *H. armigera, Pieris brassicae, Plutella xylostella, and Spodoptera litura* under laboratory conditions and are good candidates for commercialization. A number of strains have been tested and found effective against a wide range of lepidopteran, coleopteran, dipteran, and homopteran pests, gaining considerable attention.

### 2.2. *Bacillus thuringiensis* subsp. *israelensis* (*Bti*)

In India, 12 *Bt*-based products, including mosquito, black fly, and fungus gnat larvae, are registered to be used against dipteran pests. In many parts of India, Bt plays a vital role in controlling a variety of human disease vectors (Amalraj et al., 2000; Poopathi et al., 2014). The efficiency of these products usually depends on the strain and target, although they are effective under specific circumstances. Bactoculicide sprayed at 0.5 g/m^2^ (5 kg/ha) reduces *Aedes aegypti* and *Aedes albopictus* mosquito larvae by >90% inbreeding habitats *and Culex quinquefasciatus* in drains (Mittal, 2003). Despite up to the 150-fold field resistance recorded in treated field populations of the *Culex pipiens* complex, commercial formulations of the related species *Lysinibacillus sphaericus* are used to a limited extent in mosquito control programs in India (Poopathi et al., 2015). These products are being applied to manage the dipterans, especially hematophagous mosquitoes.

### 2.3. Mode of action

**Figure 2:**
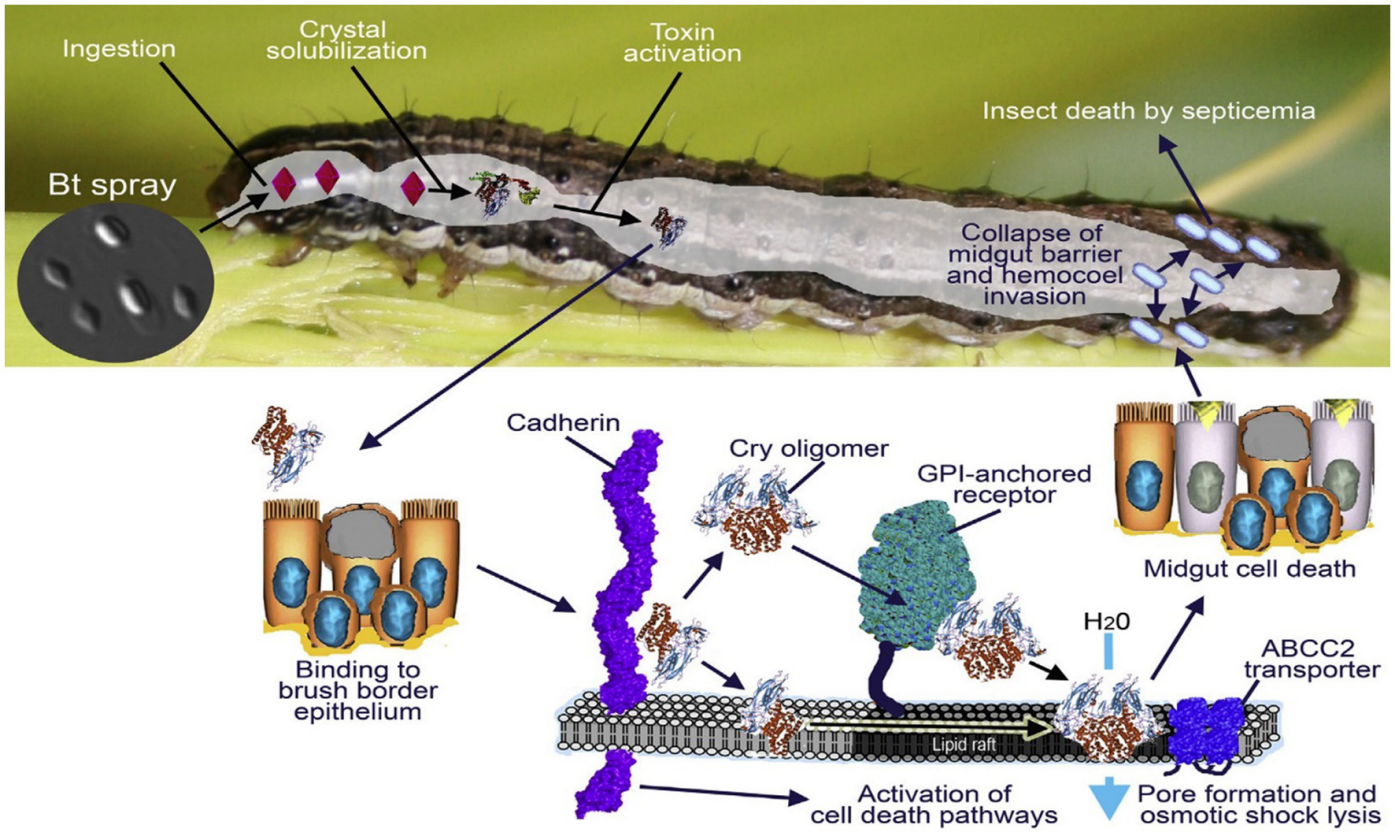
Graphical illustration of *Bt* Cry toxin and its impact on the host, after the ingestion crystal reaches the midgut and the toxin becomes activated, causing mortality; the figure was adapted with permission from Glare et al. (2017). Copyright © 2017, All rights are reserved.

Bacillus contains Cry toxin, which primarily affects lepidopteran insects. The insect gut with high pH or alkalic conditions activates the delta-endotoxin and becomes attached to the midgut receptors. Furthermore, it generates pores in midgut cells, due to which the cell loses its fluids; consequently, the midgut epithelial cell lyses after the midgut paralyzes. The cell fluids become mixed with the hemocoel and hemolymph of the insect. The pH level become imbalanced, resulting in septicemia and insect mortality. This is a direct action of the cry toxin (De Maagd et al., 2001) (Figure 2).

Nowadays, several researchers are focusing on entomopathogenic bacteria that can be effectively used in different integrated pest management strategies (Table 1).

**Table 1:** Different strains of bacterial products are commercially available worldwide against target species.

**SN**	**Entomopathogenic bacteria**	**Target pest**	**References**
1.	*Brevibacillus laterosporus*	*Musca domestica*	Ruiu et al., 2008 Ruiu et al., 2011 Zimmer et al., 2013
		Dipteran sp. *Anthonomus grandis Anticarsia gemmatalis Musca domestica*	Bedini et al., 2020 Justo De Oliveira et al., 2004
		*Aedes aegypti*	Ruiu et al., 2008
2.	*Bacillus sphaericus*	*Musca domestica Melolontha Anopheles gambiae*	Zimmer et al., 2013 Sezen et al., 2007 Fillinger et al., 2003
3.	*Bacillus thuringiensis*	*Melolontha melolontha Phyllocnistis citrella Callosobruchus maculatus*	Sezen et al., 2007 Dias et al., 2005 Malaikozhundan and Vinodhini, 2018
		*Tribolium castaeum Leucinodes orbonalis*	Elgizawy and Ashry, 2019 Tripura et al., 2017
		*Earias vittella*	Akter et al., 2017 Fourie et al., 2017 Mahapatro and Gupta, 1998 Al-Azawi, 1964; El Husseini, 2020 Ali K. et al., 2015
4.	*Bacillus weihenstephanensis*	*Melolontha melolontha*	Sezen et al., 2007
5.	*Bacillus cereus*	*Earias vittella*	Thontadarya et al., 1975
6.	*Bacillus*. *thuringiensis* var. *Kurstaki*	*Musca domestica Plutella xylostella Helicoverpa armigera*	Zimmer et al., 2013 Legwaila et al., 2015 Abedi et al., 2014
7.	*Bacillus thuringiensis* var. *israelensis*	*Musca domestica; Melolontha melolontha Anopheles gambiae*	Zimmer et al., 2013 Sezen et al., 2007 Fillinger et al., 2003
8.	*Lysinibacillus sphaericus*	*Aedes aegypti Culex quinquefasciatus*	Rojas-Pinzon and Dussan, 2017 Santana-Martinez et al., 2022
9.	*Chromobacterium subtsugae*	*Nezara viridula Diabrotica* spp.	Martin et al., 2007
10.	*Clostridium bifermentans* var. *malaysia*	*Aedes aegypti Culex pipiens, Anopheles stephensi*	Barloy et al., 1996 Qureshi et al., 2014
11.	*Yersinia entomophaga*	*Wiseana* spp. *Scopula rubraria Eucolaspis* sp.	Hurst et al., 2020 Jones et al., 2015 Hurst et al., 2011
12.	*Yersinia* spp.	*Locusta migratoria*	McNeill and Hurst, 2008
13.	*Paenibacillus Popillia*	*Popillia japonica*	Glare et al., 2017
14.	*Serratia marcescens*	*Glossina morsitans Glossina pallidipes Heliothis virescens*	Poinar Jr et al., 1979
		*Pieris brassicae Yponomeuta malinellus Neodiprion sertifer*	Sikorowski et al., 2001
		*Malacosoma Neustria Hyphantria cunea Euproctis chrysorrhoea Curculio elephas*	Sezen et al., 2001
15.	*Burkholderia* spp.	*Riptortus pedestris Spodoptera exigua*	Kil et al., 2014
		*Tetranychus urticae*	Cordova-Kreylos et al., 2013
16.	*Streptomyces* spp.	*Spodoptera litura*	Kaur et al., 2014

### 2.4. Mode of action of *Bt* in causing insect mortality

Insect mortality caused by *Bt* cotton has been intensively investigated over the last few decades, and several recent studies offer new insights into the mechanism of action.

The mechanism of action of the *B. thuringiensis* toxin Cry1Ac in the cotton bollworm (*H. armigera*) was examined by Zhang et al. (2021). They determined that the toxin alters the proportion of amino acids in the insect's midgut, increasing the number of deadly dipeptides that ultimately result in the death of gut cells and insect mortality. In addition to generating oxidative stress in cotton bollworm larvae, a study by Yao et al. (2020) showed that the Cry1Ac toxin may also cause mitochondrial malfunction, which leads to insect mortality.

Zheng et al. (2019) discuss how the insect gut microbiota affects the toxicity of *Bt* toxins such as Cry1Ac. They suggest that interactions between *Bt* toxins and the gut microbiota can reduce the toxin's toxicity and enable certain insects to develop resistance to it.

These investigations demonstrate the intricacy of *Bt* cotton's method of producing insect mortality and conclude that the Cry protein may kill insects through various channels. Researchers can create measures to maximize the efficacy of Bt cotton and reduce the possibility that resistance may emerge over time by comprehending these mechanisms.

## 3. Efficient entomopathogenic fungi against devastating pests

Entomopathogenic fungi are parasitic microorganisms that can infect and kill insects. They are mainly employed as biopesticides in ecological farming as a non-toxic alternative to toxic chemical insecticides, while some are used in biotechnological processes (Jaihan et al., 2016; Ríos-Moreno et al., 2016; Lovett and St. Leger, 2017). Generally, they have immobile chitinized cells (Badii and Abreu, 2006). Entomopathogenic fungi do not belong to a single phylogenetic group. To date, 12 Oomycetes species, 65 Chytridiomycota species, 339 Microsporidia species, 474 Entomophtoromycota species, 238 Basidiomycota species, and 476 Ascomycota species have been reported (Araújo and Hughes, 2016). Several biopesticides based on Metarhizium, Beauveria, Paecilomyces, Isaria, and Lecanicillium species are being applied worldwide. Because all these fungi have a broad range of action, they can infect a large range of arthropod species (Khan et al., 2012; Castro et al., 2016; Ríos-Moreno et al., 2016). They attack many insect and mite species, but particular species and fungal strains are distinct. Depending on the species of fungus and the number of infecting spores, host death might take 4–10 days. When the host dies, the fungus generates thousands of new spores on the corpse, spreading and completing the life cycle of new victims.

*Beauveria bassiana* was identified as the source of the destructive muscardine infection of silkworms by Agostino Bassi in 1835. It was essential in establishing the germ theory of disease. Elie Metchnikoff was among the first to suggest feasible microbial management of an insect crop pest in 1880 when he began testing the fungus *Metarhizium anisopliae* against grain beetles (Lord, 2005). In India, several research institutes are conducting experiments to determine entomopathogens' efficacy, including NBAIR in Bengaluru, Indian Institute of Oilseed Research (IIOR), Hyderabad, Central Plantation Crops Research Institute (CPCRI), Kayamkulam, and University of Agricultural Sciences, Dharwad. To date, studies have been conducted on species *B. bassiana, Metarhizum anisoplie, Lecanicillium lecanii, Nomuraea rileyi*, and *Hirsutella* spp. (Ramanujam et al., 2014).

### 3.1. *Beauveria* species

*Beauveria bassiana* has been extensively studied on crops against a wide range of lepidopteran, coleopteran, and hemipteran pests. Commercial formulations are available in aqueous suspensions, wettable powder (WP), and talc, with recommended application rates ranging from 107 to 108 conidia/ml and 400 to 750 lit/ha, depending on the crop. Sahayaraj and Namachivayam (2011) observed that an oil-based formulation of *B. bassiana* revealed 72% mortality of the larval population of *S. litura*, and a significantly higher yield was found in Tamilnadu. The suspension of commercial product (Daman 1% WP) demonstrated a potential impact on reducing *H. armigera* and enhanced sunflower seed yield (Kumar and Kaur, 2017). Recent studies indicated the potential efficacy of *B. bassiana* against the sugarcane internode borer, *Chilo Sacchariphagus indices*, and do not cause adverse effects on natural enemies.

The application of *B. bassiana* demonstrated the preservation of parasitoids of *Chilosacchariphagus indicus* (Ramasubramanian et al., 2014; Srikanth et al., 2016). Another study found that an isolate of *B. bassiana* at 4 gm/l from the Indian Institute of Horticultural Research (IIHR) had shown 81% reduction in fruit infestation caused by the tea mosquito bug, *Helopeltis antonii* on guava (NBAIR, 2017). Beauveria-based commercial products have been widely used to suppress white grubs (scarabids) in sugarcane for several years; their application facilitated the semi-perennial crop environment and limited the efficacy of conventional pesticides. Visalakshi et al. (2015) found that applying commercial talc-based *B. bassiana* formulations (5 × 10^13^) treated with FYM to the soil diminished the white grub *Holotrichia consanguinea* and damaged the sugarcane by 88% as compared with the control. Due to its long-term durability in treated fields and effectiveness against scarab, *Holotrichia serrata*, a related species, *B. brongniartii*, has emerged as a possible biocontrol agent in sugarcane (Srikanth et al., 2010). Another study advocated that the soil-based application of formulation *B. brongniartii* (2.5 kg/ha) improved yield under field conditions in Tamilnadu, India (Chelvi et al., 2011).

### 3.2. *Metarhizium anisopliae*

In India, ~30 products based *on M. anisopliae* are used to combat foliar and soil-inhabiting pests, primarily in areca nut, coconut, coffee, corn, potato, pigeon pea, and sugarcane crops. These formulations have offered a remarkable suppression of devastating insect pests in several cases. Previous experimental results revealed that a talc-based formulation of *M. anisopliae* (5 × 10^13^ spores/ha) supplemented with FYM suppressed white grub damage to sugarcane by 93% and grub population by 77% over 2 years (Visalakshi et al., 2015). Treatment applied at the time of planting revealed spectacular success compared to late application. A fungal formulation combined with FYM and sprayed to the root zone at 4 × 10^9^ conidia/ha reduced grubs by 92%. *Metarhizium* substances are utilized to keep lepidopteran pests away from tuber crops. Pandey (2013) noted the efficacy of fungal formulations and recommended applying with compost (5 × 10 spores/gm) in potatoes to protect the tuber from cutworm, *Agrotis ipsilon* attack.

### 3.3. *Lecanicillium lecanii*

More than 60 products based on *L. lecanii* (still registered under the species Verticillium) are made in liquid and dry formulations in India to regulate aphids, scales, and other soft-bodied sucking pests on a range of crops. Several recent studies attest to their efficiency against pests, including thrips and mealybugs, especially when treated at 10^8^ CFU/ml and in combination with other biopesticides (Annamalai et al., 2016; Halder et al., 2018). *Lecanicillium lecanii* is one of the most potent fungal products against aphids. According to Ramanujam et al. (2017), among 10 isolates of entomopathogenic fungi evaluated in field plots in Karnataka, *L. lecanii* VI-8 proved to be highly effective against cowpea aphid, *Aphis craccivora* (78% decrease at 10^8^ CFU/ml) and boosted yields by 32%. Similar investigations were done and observed in field conditions that *L. lecanii* at 10^9^ resulted in 72% mortality of cowpea aphids (Suresh et al., 2012).

### 3.4. *Isaria fumosorosea*

The entomopathogenic fungi, *Isaria farinosa* and *Isaria fumosorosea*, were known as *Paecilomyces farinosus* and *Paecilomyces fumosoroseus*, respectively, for more than 30 years. Both fungi have a worldwide distribution and a relatively wide host range. While *I. farinosa* currently is of minor importance in research and as a biocontrol agent, *I. fumosorosea is* regarded as a species complex, and various strains are successfully used for the biocontrol of several pest insects, mainly whiteflies (Luangsa-Ard et al., 2005). They can initiate epizootics under natural field conditions. This fungus is used as a potential biocontrol agent against invasive pests of India *viz., Aleurodicus rugioperculatus* and Bondar's nesting whitefly, *Paraleyrodes bondari* on coconut (Ali A. D. et al., 2015; Kumar et al., 2016), Ficus whitefly, *Singhiella simplex* on Ficus (Avery et al., 2019). *Isaria fumosorosea* also showed high pathogenicity (80.65%) on fourth-instar nymphs (pupae), leading to a drastic reduction of adult emergence that may result in less perpetuation of the pest in the coconut ecosystem. In addition, high mycosis was also observed in newly emerged adults. The percentage mortality of different stages of *A. rugioperculatus* significantly increased with increased spore concentrations. Findings indicate that a higher concentration of fungal spores resulted in faster control of the targeted pest (Kumar et al., 2018).

### 3.5. *Nomuraea rileyi*

*Nomuraea rileyi* infects many lepidopteran larvae, such as *S. litura, Spodoptera exigua, H. armigera, Helicoverpa zea, Trichoplusia ni, Plusia* sp., and various noctuid defoliators. When a favorable condition of humidity (>70%) and temperature (20–30°C) exist for a long time, *N. rileyi* is known to cause natural epizootic in larval populations of *S. litura, H. armigera*, and *Plusia* sp. on castor, cotton, groundnut, red gram, and niger in Andhra Pradesh. Colonies of the fungus are white initially and later turn pale green to malachite green. Hyphae are 2–3 μm in diameter, smooth, septate, hyaline, and slightly pigmented. Conidiophores are long (160 μm) and consist of dense compacted clusters of phialides and branches in whorls on the upper section. The branches are short and swollen. Phialides are short and cylindrical to globose, with a very swollen base tapering abruptly to a narrow neck. Conidia are produced in divergent dry chains on phialides, elliptical to cylindrical, measure 3–4 × 2–2.5μm, and are pale green. The fungus sporulates well only on specific media such as Sabouraud Maltose Agar with yeast (SMYA). Crushed sorghum grain supplemented with 1% yeast extract was used to mass-produce *N. rileyi*. A maximum of 1.4 × 10^9^ conidia/g of *N. rileyi* was obtained after 8–9 days at 25°C on this medium (Devi, 1994). Carrot agar supplemented with 1% yeast extract was found suitable for culturing and producing *N. rileyi*. Compatibility tests of *N. rileyi* with plant extracts and vegetable oils were conducted against *S. litura*. None of the vegetable oils were detrimental to the *N. rileyi* grown on crushed sorghum, but they were found effective in the field control of *S. litura* on castor and groundnut when applied as a foliar spray (2 × 10^11^ conidia/l) or as soil treatment at 10g/m^2^ (2 × 10^11^ conidia/g). It controls pod borers, cutworms, and cabbage borers.

### 3.6. *Hirsutella thompsonii*

These fungi control different hoppers, bug pests, whiteflies, and red mites. It is a promising candidate for the control of citrus red mites and coconut eriophyid mites. It is a synnamatous fungus with bulbous phialides arising laterally from synnema or the hyphae. The conidia are one-celled and hyaline. In India, the fungus has been isolated from the eriophyid mite, *Aceria guerreronis*, and tested for control of same (Table 2).

**Table 2:** Toxins produced by entomopathogenic fungi.

**Fungi**	**Toxin**
*Cordyceops miliaris*	Cordycepin
*Beauveria bassiana*	Beauvericin
*B. Brongnihartii*	Beauverolides, Bassinolidae
*Metarhizium anisopliae*	Isarolides A, B, C, D
*Paecilomyces* spp.	Beauvericin
*Isaria* spp.	Isarin
*Verticillium*	Similar to Bassinolidae

### 3.7. Mode of action on the target insect

**Figure 3:**
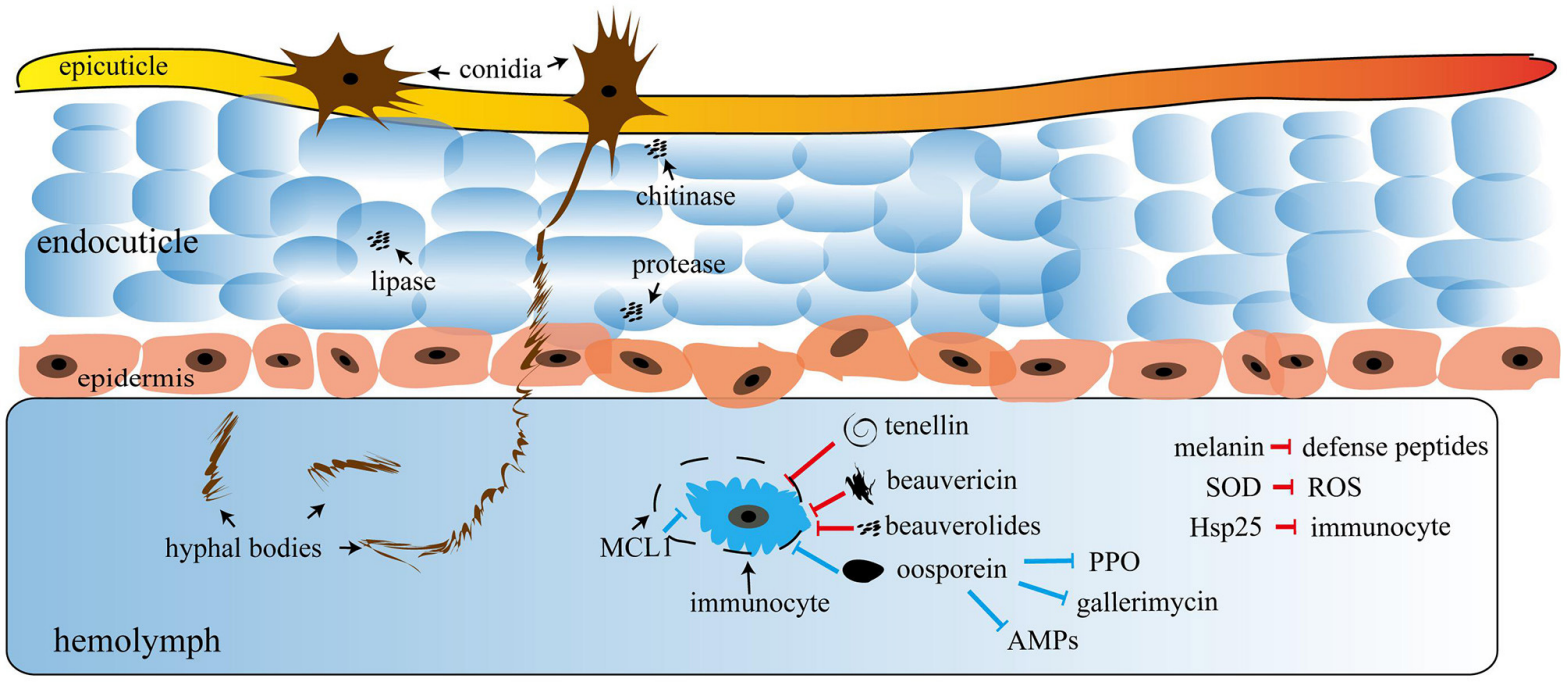
Mechanism of entomopathogenic fungi (EPF); the figure was adapted with permission from Wang H. et al. (2021). Copyright © 2021 Wang, Peng, Li, Cheng and Gong.

The fungus develops specialized structures to penetrate into the host integument, such as appressoria, which allows the developing hyphae to enter into the host integument (Ortiz-Urquiza and Keyhani, 2013). Some enzymes, such as metalloid proteases and aminopeptidases, perform cuticle degradation through germ tubes (Bidochka and Small, 2005). The fungus hyphae disseminate inside the insect hemocoel and attack various muscle tissues, fatty bodies, Malpighian tubes, mitochondria, and hemocytes; as a result, infected insects die after some days (Kachhawa, 2017). The Hyphomycetes genus kills insects through nutritional deprivation, tissue damage, and producing toxins in the insect body. Entomopathogenic fungus and cuticle degrading enzymes, such as chitinase, protease, and lipase, play a vital role in the pathogenicity in insects because they dissolve the cuticle to enter the germ tube insect's body (Figure 3).

Entomopathogenic fungi enter insects through two processes: the first is mechanical pressure on the cuticle, and the second is enzymatic degradation. At the time of pathogenesis, several mycotoxins are produced, including Beauvericin (*B. bassiana*), Beauverolides (*Verticillium lecanii*), Bassianolide (*Paecilomyces* spp.), and Destruxins A, B, C, D, E, F (*M. anisopliae*), which are poisonous to the insects. After the insect's death, fungus emerges from the integument, produces aerial mycelia, and sporulates on the cadavers. Some Hypoclealean fungi, such as *B. bassiana, I. fumosorosea, Hirsutella thompsonii, L. lecanii, Metarhizium acridum, M. anisopliae*, and *M. brunneum*, are commercially available as biopesticides in a variety of formulations all over the world. These are effective against piercing and sucking mouthparts and pests with chewing mouthparts. The entomopathogenic fungus obligates insects that kill their hosts by colonizing their tissues without toxins (Pell et al., 2001). Natural epizootics of entomophthoralean fungi, such as *Entomophaga maimaiga, Entomophthora muscae, Neozygites fresenii, Neozygites floridana*, and *Pandora neoaphidis*, are known to cause a massive decrease in the host population. Even though such delicate fungi are challenging to subculture under artificial conditions, they may be crucial in the natural control of particular pest species (Table 3).

**Table 3:** List of entomopathogenic fungi effective against different harmful pests.

**SN**	**Entomopathogenic fungi**	**Target pest**	**References**
1.	*Beauveria bassiana*	*Lipaphis erysimi Schizaphi graminum, Rhopalosiphum padi*,	Ujan et al., 2012 Gul et al., 2015
		*Brevicoryne brassicae Myzus persicae Leucinodes orbonalis Earias vittella Aphis craccivora Sitophilus granaries*	Michereff Filho et al., 2011 Tripura et al., 2017 Mathur et al., 2012 Ali K. et al., 2015 Batta, 2018 Saranya et al., 2010
		*Dicladispa armigera Helicoverpa armigera*	Sharma et al., 2017 Devi and Hari, 2009
	*Beauveria brongniarti*	*Holotrichia serrata*	Chelvi et al., 2011
2.	*Lecanicillium lecanii* (*Verticillium lecanii)*	*Lipaphis erysimi Leucinodes orbonalis Brevicoryne brassicae Myzus persicae Aphis craccivora Lipaphis erysimi*	Ujan et al., 2012 Mathur et al., 2012 Asi et al., 2009 Khan and Khalil, 1990 Saranya et al., 2010 Rana et al., 2002
3.	*Paecilomyces lilacinus*	*Lipaphis erysimi Brevicoryne brassicae*	Ujan et al., 2012 Asi et al., 2009
4.	*Metarhizium anisopliae*	*Lipaphis erysimi Leucinodes orbonalis Brevicoryne brassicae Aphis craccivora Melolontha melolontha Sitophilus granaries Acyrthosiphon pisum Tetranychus urticae Tetranychus cinnabarinus Helicoverpa armigera Holotrichia longipennis*	Ujan et al., 2012 Tripura et al., 2017 Asi et al., 2009 Saranya et al., 2010 Putnoky-Csicso et al., 2020 Batta, 2018 Seye et al., 2014 Mariam et al., 2016 Nahar et al., 2010 Pandey, 2010
5.	*Aspergillus flavus*	*Earias vittella Acyrthosiphon pisum*	Shitole and Patel, 2010 Seye et al., 2014
6.	*Hirsutella thompsonii*	*Aphis craccivora Mononychellus tanajoa Tetranychus neocaledonicus*	Saranya et al., 2010 Odongo et al., 1998 Rachana et al., 2015
8.	*Cladosporium cladosporioide*	*Tetranychus urticae Tetranychus cinnabarinus*	Habashy et al., 2016
		*Tetranychus urticae*	Saranya et al., 2013
9.	*Aspergillus clavatus*	*Acyrthosiphon pisum*	Seye et al., 2014
10.	*Fusarium semitectum Berk*	*Tetranychus neocaledonicus*	Rachana et al., 2015
11.	*Nomuraea rileyi*	*Spodoptera litura Thysonoplusia orichalcea Helicoverpa armigera*	Rachappa et al., 2007

## 4. Compatibility of entomopathogenic fungi with other biological agents

As part of integrated pest management (IPM) techniques, there is increased interest in combining entomopathogenic fungi with other biological control agents. The compatibility of entomopathogenic fungi with other biological agents, including parasitoids, predators, and other microbes, has been the subject of some recent investigations. Here are a few current study examples: Wei et al. (2021) investigated the compatibility between the parasitoid wasp, *Habrobracon hebetor*, employed to manage several moth pests, and the entomopathogenic fungus, *B. bassiana*. They observed that the two plants, *B. bassiana* and *H. hebetor*, worked better together than alone to combat the moth pest, *Plodia interpunctella*. In research by Dubovskiy et al. (2020), the predatory mite *Amblyseius swirskii* and the entomopathogenic fungus *Metarhizium robertsii* were used to suppress the tomato russet mite (*Aculops lycopersici*). The scientists discovered that *M. robertsii* and *A. swirskii* worked synergistically to reduce the tomato russet mite population significantly. To manage the autumn armyworm (*Spodoptera frugiperda*), Wang B. et al. (2021) studied the compatibility of the entomopathogenic fungus *I. fumosorosea* with the bacterium *B. thuringiensis*. They determined that *I. fumosorosea* and *B. thuringiensis* worked together to suppress the autumn armyworm more effectively than each of them did by itself.

These studies highlight how entomopathogenic fungus may be applied with other biological pest control agents to boost pest control effectiveness and decrease dependency on chemical pesticides. However, the compatibility of various biological control agents in contexts of several pest management needs additional investigation.

## 5. Entomopathogenic viruses

An entomopathogenic virus is a tiny, essential infectious agent of nucleic acid (RNA or DNA) encased in a protective protein coat known as the capsid, which can multiply in a susceptible host cell. If a lipid bilayer envelops the nucleocapsid, it is a virion. The word virus means “poison,” derived from a Latin term.

Mathews and Shenk (1991) defined a virus as “a set of one or more nucleic acid template molecules, generally encased in a protective coat or coats of protein or lipoprotein, that can organize its replication only within suitable host cells.” Within such cells, virus replication is (i) dependent on the host's protein-synthesizing machinery, (ii) organized from pools of the required materials rather than by binary fission, (iii) located at sites that are not separated from the host cell contents by a lipoprotein bilayer membrane, and (iv) continually giving rise to variants through various kinds of change in the viral nucleic acid.

The virus nucleic acid (DNA or RNA) is crucial to the infection of the host cell and drives viral replication. These are classified into two genera, nucleopolyhedroviruses (NPVs) and granuloviruses (GVs). Both genera have a circular double-stranded DNA genome of ~80–180 kb packed within rod-shaped nucleocapsids and anticipated to encode 90–180 genes (Okano et al., 2006). Nucleopolyhedroviruses have polyhedral OBs, made of crystalline polyhedrin protein, which occludes massive virions. In contrast, granuloviruses have smaller granule-like OBs, made up of granulin protein. Every granulovirus OB contains a single virion. The Baltimore classification used to classify viruses is based on nucleic acids and capsid function (Sparks et al., 2010). When a virus enters a compatible host cell, its nucleic acid takes control of the metabolic process. It multiplies rapidly, producing new virus particles until the cell is deprived of its contents and dies; thus, a virus is classified as an obligatory parasite since it consumes the cell's material and its metabolic processes. Viruses cannot multiply “*in vitro*.” Although all viruses have the same fundamental structure and require host cells to reproduce, viruses in nature come in various shapes and sizes. All viruses have different morphologies, genomes, infectivities, and host ranges. Entomopathogenic viruses are both a potential and essential part of an integrated pest control approach. The few families of viruses show infectivity against the insects.

Moreover, entomopathogenic viruses belong to the Baculoviridae family. That family infests some orders, such as lepidoptera, hymenoptera, and diptera, as a natural host. These entomopathogenic viruses are managed by various agricultural and forest insect pest populations worldwide as biopesticides (Sun and Peng, 2007). Viruses are being used as a promising tool for managing economically important insect pests; various viruses, viz. nucleopolyhedrovirus (NPV), granulosis viruses (GVs), and cytoplasmic polyhedrosis viruses, are used for the management of insect pests throughout the world. The insects ingest the host-specific viral particles, and the virions infect the gut wall cells, fat body, and hemolymph, leading to the death of the insects. As a result, their application is innovative and environmentally friendly. They are non-hazardous to human health and the environment, offering novel industrial products and field uses (Sumathy et al., 1996). Baculoviruses have a single circular genome surrounded by rod-shaped nucleocapsids. To produce virions, enveloped by membrane singly or in groups, occluded in a protein matrix, forming the occlusion body (OB) (Williams et al., 2017).

### 5.1. General features of insect viruses

Insect viruses belong to many different virus families, some of which occur exclusively in arthropods and some of which include representatives in vertebrates and/or plants. A feature of many insect viruses, which does not occur in viruses infecting plants or vertebrates, is that they are occluded, i.e., the virions are embedded within a proteinaceous body. Occlusion bodies (OBs) vary in size from about 0.5 to over 20 μm across but are all visible under the light microscope.

### 5.2. Application of NPV on the target pest

*Helicoverpa armigera* granulosis virus is known as HearGV, while the *Autographa californica* multiple nucleopolyhedroviral is known as AcMNPV. As a result, all nuclopolyhedrovirus is known as NPV, whereas granulosis viruses are GV. In managing *H. armigera* and *H. zea* populations that have gained resistance to chemical pesticides, NPV-based products are an important alternative to chemical pesticides (Kranthi et al., 2002). HaNPV is applied at 250 LE/ha (1.5 × 10^12^ POBs/ha) according to the author's recommendation (Saxena and Ahmad, 2005; Srinivasa et al., 2008). In the field trials, the HaNPV CBE-1 isolate reduces the *H. armigera* by 64 and 62% in cotton chickpeas, respectively, compared to untreated plots (Jeyarani et al., 2010). While we are uninformed of any granuloviruses (GV) available on the market in India, research conducted with native isolates has shown that GV might be employed to effectively tackle the sugarcane shoot borer *C. infuscatellus* (Rabindra, 2005; Rao and Babu, 2005). It is also noticed that it effectively suppresses the diamondback moth, *P. xylostella* (Jayanth, 2002).

The table shows the entomopathogenic virus and its target insect host, tested and found effective in causing insect mortality (Table 4).

**Table 4:** Potential NPV against insect pests.

**SN**.	**Entomopathogenic virus**	**Target host**	**References**
1.	Nuclear Polyhedrosis Virus (NPV)	*Spodoptera frugiperda Spodoptera litura*	Barrera-Cubillos et al., 2017 Maqsood et al., 2017 Kumari and Singh, 2009
		*Galleria mellonella Helicoverpa armigera*	El Husseini, 2020 Nawaz et al., 2020 Wakil et al., 2012 Jagadish et al., 2010 Dhandapani et al., 1993 Mir et al., 2010 Lopes et al., 2020
		*Anticarsia gemmatalis*	
		*Chrysodeixis includens Ostrinia nubilalis Trichoplusia ni Hyblaea puera Earias vittella*	Lewis and Johnson, 1982 Hostetter et al., 1979 Nair et al., 1996 Khan et al., 2019
2.	Granulovirus (GV)	*Cydia pomonella*	Jaques et al., 1987 Schmidt et al., 2008
		*Plodia interpunctella*	Vail et al., 1991 McGaughey, 1975
		*Pieris rapae Chilo infuscatellus*	Tatchell and Payne, 1984 Easwaramoorthy and Santhalakshmi, 1988
		*Phthorimaea operculella Pieris brassicae Agrotis segetum*	Mascarin and Delalibera, 2012 Peters and Coaker, 1993 Zethner, 1980

### 5.3. Mechanism of NPV

**Figure 4:**
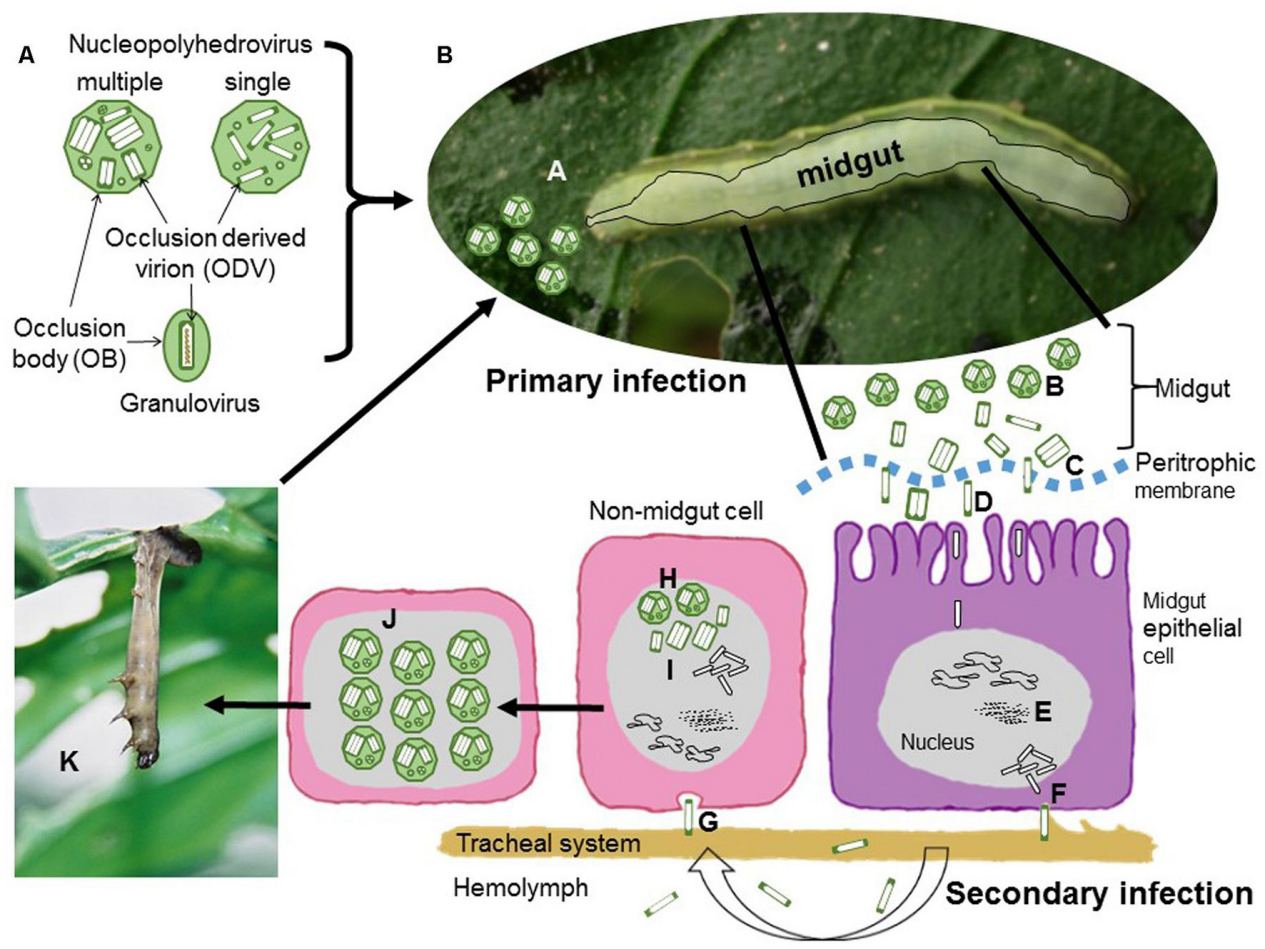
**(A)** Nucleopolyhedrovirus occlusion bodies (OBs) are polyhedral proteinaceous structures that surround occlusion derived virions (ODVs). Each ODV contains either a single nucleocapsid (single type) or one to several nucleocapsids (multiple type). Granulovirus OBs are granule-shaped and contain a single ODV with a single nucleocapsid encased in the crystalline protein granulin. Each nucleocapsid carries a single viral genome in all situations. **(B)** The processes of nucleopolyhedrovirus transmission and replication. During initial infection, **(A)** OBs are ingested while feeding on infected vegetation later on OBs are solubilized in the insect midgut, releasing ODVs that pass through the peritrophic membrane and merge with the microvilli of midgut epithelial cells. Afterwards, Nucleocapsids migrate to the nucleus and release the viral DNA to begin replication. These budded virions (BVs) diffuse in the hemolymph or along the cells of the insect traqueal system (traqueoblasts) during the secondary phase of infection, spreading the infection to the cells of different tissues in the insect.

The baculovirus is transmitted orally. The entomopathogenic virus spreads by ingesting infested food from insects, such as bacterial infection in insects. Because these are suitable for controlling insects. The virus enters the body of insects by infesting food. The occlusion bodies get dissolved in the midgut; virus particles invade the midgut epithelium, fat body, and other tissue cells, damaging the tissue integrity and dissolving the cadavers. Before death, infected larvae climb the upper portion of the plant, allowing virus particles from the cadavers to the lower parts of the plant. This behavior allows the virus to spread to cause infection in healthy larvae. Viruses are very host-specific and may cause a significant reduction in host populations (Figure 4).

Due to host specificity, entomopathogenic viruses are essential to control insects and pests. Several virus-based commercial biopesticides are available in markets and have demonstrated better results than other control practices. Many researchers found that entomopathogenic viruses have essential characteristics and efficacy against different insects.

## 6. Entomopathogenic nematodes

Rao and Manjunath (1966) were the first to demonstrate that the *Steinernema carpocapsae* DD-136 strain was applied to target lepidopteran pests of rice, sugarcane, and apple in India. For scientific purposes, exotic strains of *S. corpocapsae, Steinernema feltiae, Steinernema glaseri*, and *Heterorhabtidis bacteriophora* were introduced later (Rahaman et al., 2000). Subsequently, extensive surveys were carried out to find out potent indigenous EPN species *S. carpocapsae* (Hussaini et al., 2001), *Steinernema abbasi* (Hussaini et al., 2003), *Steinernema bicornutum* (Hussaini et al., 2001), *Steinernema thermophilum* (Sudershan and Singh, 2000), *Steinernema tami* (Hussaini et al., 2001), and *Steinernema riobrave* (Ganguly et al., 2002). The product (Pusa Nemagel) has a long shelf life and inhibits white grubs, termites, and a variety of other lepidopteran pests (Ganguly et al., 2008). *Steinernema thermophilum* was the first EPN to be identified as pathogenic to lepidopteran eggs (Kalia et al., 2014). In Uttarakhand and Uttar Pradesh, large-scale EPN was tested in response to persistent white grub infestations in sugarcane. The Indian Agriculture Research Institute (IARI, New Delhi) undertook a biocontrol initiative, including the mass breeding and release of EPN-infested insect cadavers to counter the infestation (Mohan et al., 2017). Farmers were trained on *in vivo* rearing and field application as part of the research, which designed a low-cost EPN rearing method in *Galleria mellonella*.

### 6.1. Bacteria associated with parasitic nematodes (symbiotic association)

**Figure 5:**
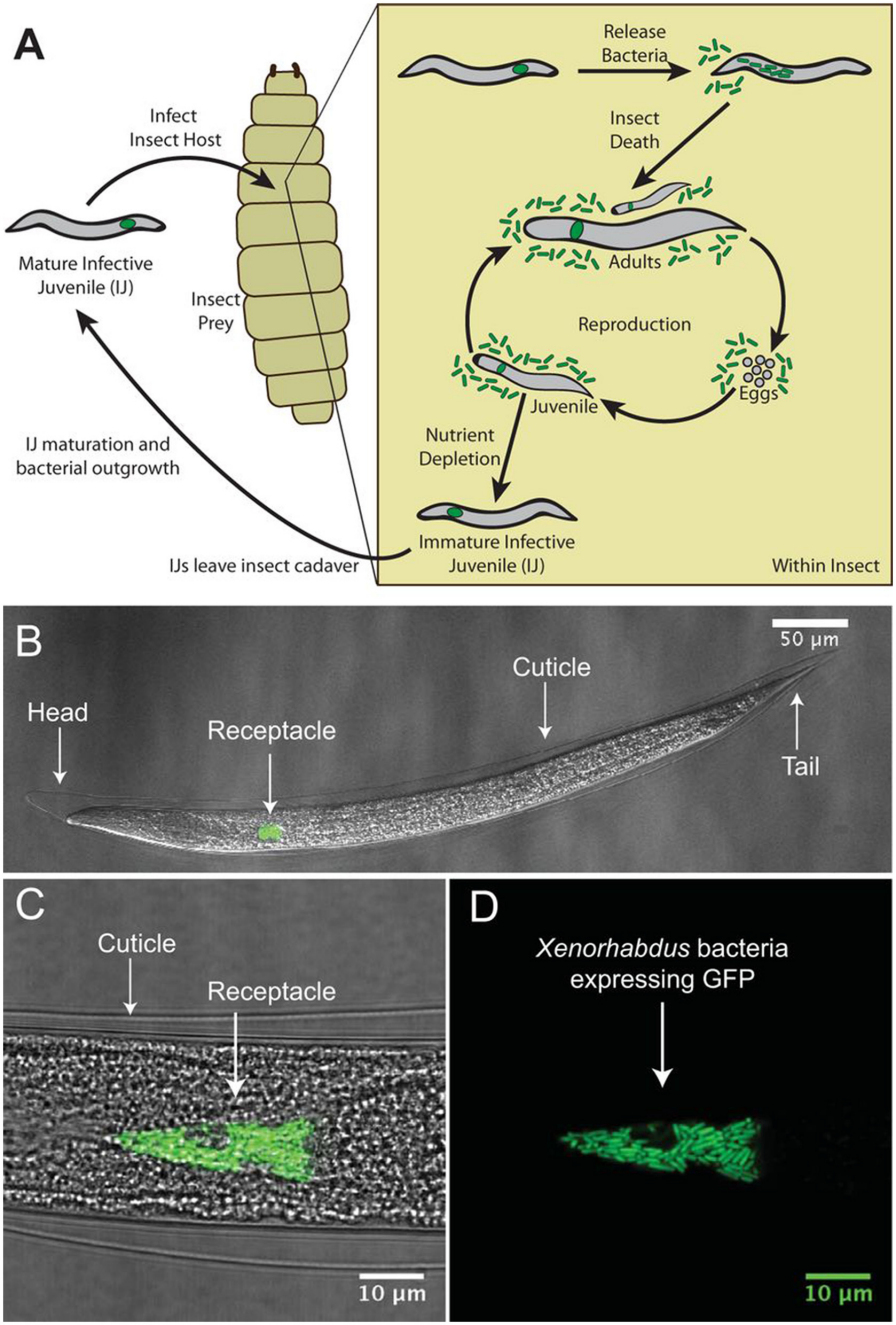
The mutualistic relationship between the bacterium *Xenorhabdus nematophila* and nematode, *Steinernema carpocapsae*. **(A)** Illustration of the tripartite life cycle of *S. carpocapsae* nematodes. Infective juveniles (IJs) infect an insect prey and release *X. nematophila* cells to elude the host immune system and kill the host. Both species reproduce using the cadaver's nutrients; when the nutrients impoverished, the two creatures recombine and enter the soil to restart the cycle. **(B–D)** Confocal micrographs of a *S. carpocapsae* nematode's IJ stage with GFP-expressing *X. nematophila* cells in the intestinal receptacle. Panel **D** shows the intravesicular structure (IVS) as the black region below the white arrow surrounded by bacterial cells.

Nematodes are tiny, transparent, and relatively basic in terms of multicellular creatures, and they occupy a variety of environmental niches and model species for investigating bacterial symbiosis (Dillman et al., 2012). Insect-parasitic and entomopathogenic nematodes (EPNs) belong to the genera Heterorhabditis and Steinernema, respectively, and are symbiotically correlated with the bacteria Photorhabdus and Xenorhabdus (Shapiro-Ilan et al., 2017) (Figure 5).

The infective juveniles (IJs) of EPNs dwelling in soil access the larval stage of their insect hosts by a natural opening (mouth, spiracle, or anus) or the delicate cuticle during the intricate life cycle. The IJs of ENPs release symbiotic bacteria into the body of their host, where they rapidly proliferate and generate secondary metabolites. Within 24–48 h, the infected insect host dies (Dowds and Peters, 2002). The bio-converted insect host is fed on by both the EPNs and the bacteria that remain in symbiosis. EPNs produce 1–2 generations of offspring in the insect cadaver. The symbiotic bacteria are consumed by the IJs, and when the food supply is reduced a new cohort of IJs carrying the symbiotic bacteria emerges from the cadaver in search of a new insect host. Therefore, EPNs have been employed as biocontrol agents for a variety of insect pest species (Lacey et al., 2015). Important biotic and abiotic factors are essential for EPNs' survival in ecological niches. For the survival of EPNs, soil characteristics, including texture, pH, moisture, and temperature, are particularly crucial. The variety and distribution of EPNs are affected by geographical regions, ecosystems, and soil characteristics. Variable recovery rate has been investigated in a number of surveys of EPNs from numerous geographical ecologies. Studies were carried out to investigate the interaction between Steinernema nematodes and their associated bacteria.

Several attempts have been attempted to harness native EPNs to combat insect pests notably the Caribbean fruit fly (*Anastrepha suspensa*) (Heve et al., 2017), termite (Al-Zaidawi et al., 2020), and housefly (Arriaga and Cortez-Madrigal, 2018) (Tables 5, 6).

**Table 5:** Important entomopathogenic nematodes (EPNs) to manage target insect pests.

**Bioagent (nematode)**	**Target pest**	**References**
*Heterorhabditis bacteriophora, Steinernema carpocapsae*	Apple sawfly (*Hoplocampa testudinea*)	Vincent and Belair, 1992; Belair et al., 1998
*Steinernema carpocapsae, Steinernema feltiae*	Plum sawfly (*Hoplocampa minuta, Hoplocampa flava*)	NjeŽić and Ehlers, 2020
*Heterorhabtidis bacteriophora, Steinernema carpocapsae, Steinernema feltiae*	Pea sawfly (*Hoplocampa brevis*)	De Luca et al., 2015
*S. bacteriophora, Steinernema feltiae*	Whitefly (*Trialeurodes vaporariorum*)	Laznik et al., 2011; Hussain et al., 2015; Rezaei et al., 2015
*Steinernema feltiae*	Whitefly (*Bemisia tabaci*)	Cuthbertson et al., 2003, 2007; Head et al., 2004; Qiu et al., 2008
*Heterorhabtidis bacteriophora, Steinernema carpocapsae*	Cucurbit fly (*Dacus ciliates*)	Kamali et al., 2013
*Steinernema kraussei, Heterorhabtidis bacteriophora, Steinernema carpocapsae, Steinernema feltiae*	Fruit flies (*Drosophila suzukii*)	Kepenekci et al., 2015; Cuthbertson and Audsley, 2016; Garriga et al., 2020
*Steinernema carpocapsae, Heterorhabtidis bacteriophora*	Straw berry crown moth (*Synanthedon bibionipennis*)	Bruck et al., 2008
*Steinernema carpocapsae, Steinernema feltiae, Steinernema kraussei, Steinernema riobrave, Heterorhabtidis zealandica, Heterorhabtidis marelatus, Heterorhabtidis bacteriophora, Steinernema yirgalemense*	Codling moth (*Cydia pomonella*)	Unruh and Lacey, 2001; Lacey et al., 2005, 2006; Navaneethan et al., 2010; Odendaal et al., 2016; de Waal et al., 2018
*Steinernema carpocapsae*	Litchi stem borer (*Arbela dea*)	Saleh, 2017
*Heterorhabtidis bacteriophora, Steinernema carpocapsae, Steinernema feltiae, Steinernema riobrave*	Oriental fruit moth (*Cydia molesta*)	Riga et al., 2006; Negrisoli et al., 2013
*Steinernema carpocapsae*	Red longicorn beetle (*Aromia bungii*)	Liu et al., 1997; Saleh, 2017
*Steinernema bacteriophora Steinernema feltiae, Heterorhabtidis indica, Steinernema bicornutum, Steinernema carpocapsae, Thripinema nicklewoodi*	Western flower thrips (*Frankliniella occidentalis*)	Ebssa et al., 2001, 2004a,b; Wardlow et al., 2001; Belay et al., 2005; Buitenhuis and Shipp, 2005; Arthurs and Heinz, 2006; Trdan et al., 2007
*Heterorhabtidis indica, Steinernema kraussei, Steinernema feltiae*	Wheat stem sawfly (*Cephus cinctus*)	Portman et al., 2016
*Steinernema affine, Steinernema carpocapsae, Steinernema bacteriophora, Steinernema feltiae*	Tomato leaf miner (*Tuta absoluta)*	Batalla-Carrera et al., 2010; Gözel and Kasap, 2015
*Heterorhabtidis indicus, Steinernema feltiae, Steinernema bacteriophora, Steinernema carpocapsae*	Onion thrips (*Thrips tabaci*)	Al-Siyabi et al., 2006; Kashkouli et al., 2014
*Steinernema feltiae*	Melon thrips (*Thrips palmi*)	Cuthbertson et al., 2007
*Heterorhabtidis bacteriophora* BA1, *Heterorhabtidis* indica, Steinernema sp.95, *Rhabditis blumi, Heterorhabtidis bacteriophora* HNI0100	Diamondback moth (*Plutella xylostella*)	Ehlers et al., 2005; Schroer et al., 2005; Gupta et al., 2011; Johnson et al., 2012; Park et al., 2012; Hussain et al., 2015; Sáenz-Aponte et al., 2020
*Steinernema feltiae, Steinernema carpocapsae, Heterorhabtidis bacteriophora*	Carob moth (*Ectomyelois ceratoniae*)	Memari et al., 2016
*Heterorhabtidis indica* IBCB-n5, *Steinernema* splBCB-n6, *Steinernema carpocapsae, Heterorhabtidis indica, Steinernema glaseri*	textitSpodoptera frugiperda *Spodoptera litura*	Sezhian et al., 1996; Umamaheswari et al., 2006; Garcia et al., 2008
*Steinernema carpocapsae*	Brinjal fruit and shoot borer (*Leucinodes orbonalis*)	Visalakshy et al., 2009
*Heterorhabtidis indica, Steinernema karii*	Banana weevil (*Cosmopolites sordidus*)	Waturu et al., 1998
*Heterorhabtidis bacteriophora*	Caribbean fruit fly (*Anastrepha suspense*)	Heve et al., 2017
*Heterorhabtidis bacteriophora, Heterorhabtidis zealandica, Steinernema yirgalemense*	Citrus mealy bug (*Planococcus citri*)	van Niekerk and Malan, 2012; Van Niekerk and Malan, 2015; Le Vieux and Malan, 2013
*Heterorhabtidis bacteriophora, Heterorhabtidis ferruginophorus, Heterorhabtidis indica, Steinernema abbasi, Steinernema glaseri*	Red Palm weevil (*Rhynchophorus ferrugineus*)	Dembilio et al., 2010; Wakil et al., 2017; Yasin et al., 2017
*Steinernema carpocapsae, Heterorhabtidis indica*	Legume pod borer (*Helicoverpa armigera*)	Prabhuraj et al., 2004, 2005, 2008; Hussain et al., 2014; Abid and Saeed, 2015

**Table 6:** Entomopathogenic microorganisms with different commercial names against target pests.

**Active substances**	**Trade names**	**Target pests**
**Bacteria**		
*Bacillus thuringiensis aizawai*	Able-WG, Agree-WP, Florbac, XenTari, Certan	Armyworms, diamondback moth
*Bacillus thuringiensis kurstaki*	Biobit, Cordalene, Costar-WG, Crymax-WDG, Deliver, Dipel, Foray, Javelin-WG, Lepinox Plus, Lipel, Rapax	Lepidoptera
*Bacillus thuringiensis israelensis*	Teknar, VectoBac, Vectobar, Bactimos	Mosquitoes and black flies
*Bacillus thuringiensis tenebrionis*	Novodor, Trident, M-Trak	Colorado potato beetle
*Bacillus thuringiensis sphaericus*	VectoLex, VectoMax	Mosquito (Diptera)
*Burkholderia* spp.	Majestene, Venerate	Chewing and sucking pests
*Saccharopolyspora spinosa*	Tracer™ 120, Conserve	Insect pests
*Chromobacterium subtsugae*	Grandevo	Chewing and sucking pests
*Bacillus firmus*	Bionemagon	Nematodes
**Fungi**		
*Beauveria bassiana*	Bio-Power, Biorin/Kargar, Botanigard, Daman, Naturalis, Nagestra, Beauvitech-WP, Bb-Protec, Racer, Mycotrol, Conidia, Ostrinol, Biosoft, Biowonder, Myco-Jaal	Coleoptera, Lepidoptera Grasshopper, Stem borers, cutworms, leaf hoppers, whiteflies, aphids, thrips, and mealy bugs
*Beauveria brongniartii*	Bas-Eco, Betel, Engerlingspilz, Melocont	Coleoptera (Scarabaeidae), European cockchafer beetles
*Hirsutella thompsonii*	No-Mite, Mycar	Mites
*Isaria fumosorosea*	Nofly™ WP	Whitefly, aphids, thrips, psyllids, and mealy bugs
*Metarhizium anisopliae*	Achieve, Biomet/Ankush, Bio-Magic, Bio-Catch-M Kalichakra, Novacrid, Met52/BIO1020 granular, Pacer, Multiplex, Metarhizium	Mites, Aphids, whitefly, Scarabids, Isoptera
*Metarhizium brunneum*	Attracap	
*Paecilomyces lilacinus*	Bio-Nematon, MeloCon, Mytech-WP, Paecilo	
*Paecilomyces fumosoroseus*	Bioact WG, No-Fly-WP, Paecilomite, Prioroty, PFR-97	
*Verticillium lecanii*	Bio-Catch, Mealikil, Bioline/Verti-Star, Verticare, Vertalec, Mycotal, Inovert	
*Lecanicillium lecanii*	Mycotal, Vertelac	Whiteflies, Thrips, and Aphids
**Viruses**		
*HzNPV*	Gemstar	*Helicoverpa zea*
*PlxyGV*	Plutellavex	*Plutella xylostella*
*Spli NPV*	Littovir	*Spodoptera littoralis*
*eMNPV*	Spodex	*Spodoptera exigua*
*LdMNPV*	Gypchek	*Lymantria dispar*
*CpGV*	CYD-X, Madex, Carpovirusine, Granupom, Carposin Virosoft CP-4, Virin-Gyap	*Cydia pomonella*
*NeabNPV*	Neodiprion abietis NPV	Balsam fir sawfly
*SeNPV*	Spexit, Spod-X	*Spodoptera exigua*
*AcMNPV*	VPN 80	*Autographa californica*
*AgMNPV*	Polygen, multigen	*Anticarsia gemmatalis*
*OpMNPV*	TM Biocontrol	*Orgyia pseudotsugata*
*AdorGV*	Capex 2	*Adoxophyes orana*
**Nematodes**		
*Steinernema carpocapsae*	Capsanem, Carpocapsae-System, Optinem-C, NemaGard, Nemastar, NemaRed, Palma-Life	Borer beetles, caterpillars, moth larvae, *Rhynchophorus ferrugineus*, Tipulidae
*Steinernema feltiae*	Entonem, NemaShield, NemaTrident-F, Nemapom, Nemaplus, Nemaflor, Nemafly, Nematech-S SP, NemaTrident-S	Chromatomyia syngenesiae, *Phytomyza vitalbae*, soil-dwelling pests, codling moth larvae, *Thrips tabaci*
*Heterorhabtidis bacteriophora*	Larvanem, Nemaplant, NemaShield-HB, Nematop, Nematech-H, Nematrident-H	Chestnut moth, black wine weevil, soil-dwelling beetle larvae, cutworms, leaf miners

## 7. Registration and regulation

Under the Insecticides Act of 1968 and the Insecticides Rules of 1971, the Central Insecticides Board and Registration Committee (CIBRC) is the regulating authority in India that governs biopesticides. This board advises the federal and state governments on technical issues concerning the manufacture, marketing, distribution, and use of insecticides, including biopesticides, to assure human health, the environment, and natural bioagents. After evaluating their formulations and validating data on efficacy, toxicity, and packaging supplied by the importer or manufacturer, the CIBRC's registration committee permits public and private enterprises for large-scale manufacturing, distribution, and sale of biopesticides stakeholders. Manufacturers can register new products under the Insecticides Act's section 9(3B) (provision registration for a novel active component used in India) or section 9(3) (regular registration).

## 8. Challenges associated with entomopathogen registration and their solutions

Entomopathogens, which are microorganisms that may infect and kill insects, have been widely used in forestry and agriculture as biological control agents. However, there are a number of constraints in registering entomopathogens as pesticides, including lacking information on their effectiveness, safety, and environmental effects. The following are some of the major problems with entomopathogen registration and solutions.

### 8.1. Data on efficacy

The absence of effectiveness data is one of the greatest problems with entomopathogen registration. There is a need for further research to confirm the effectiveness of entomopathogens against target pests, according to a review by Zimmermann (1993). The efficiency of entomopathogens can be demonstrated by investigations and systematic reviews, as in the study by Fang et al. (2021) that assessed the efficacy of *M. anisopliae* against the maize stem borer.

### 8.2. Safety data

The absence of safety data is another challenge with entomopathogen registration. There is a need for more studies to determine the safety of entomopathogens, including their effect on non-target species, their persistence in the environment, and their potential for resistance development, according to a review by Hajek et al. (2021). Dillman et al. (2012) assessed the safety of *B. bassiana* on non-target bees, which can provide more information on the safety of entomopathogens.

### 8.3. Data on environmental effects

Another crucial factor for entomopathogen registration is their effect on the environment. Further investigation needs to be done to assess the environmental impact of entomopathogens, including their impacts on non-target species, their persistence in the environment, and their potential for bioaccumulation. Additional information on the environmental effects of entomopathogens can be found in recent research and reviews, such as the one by Hill (2020), which assessed the persistence of *B. bassiana* in soil. In order to prove their efficacy, safety, and environmental impact, more studies and systematic reviews are needed in order to address the issues associated with entomopathogen registration.

## 9. Limitation for the proliferation of microbial biopesticides

Although microbial pesticides have significant advantages over traditional pest control agents, they have failed to gain widespread commercial development and implementation in India. Some factors constrain the Indian market for microbial pesticides. Product quality control concerns, such as low microbial count, which leads to poor field performance, a lack of large-scale production facilities, and the sale of unregistered items in the market, are listed as the main hurdles (Alam, 2000; Gupta and Dikshit, 2010; Arora et al., 2011; Mishra et al., 2014). According to NBAIR studies, 50%−70% of microbial biopesticide-based products in India had deficiencies such as lesser colony propagules specified on the label, excessive moisture content in solid formulations, or pollutants. It, therefore, failed to fulfill the declared CIBRC requirement (Ramasubramanian et al., 2014). Some microbial biopesticides have a short shelf life, and there is a challenge in rural locations where fresh products and refrigerated storage are scarce (Mishra et al., 2014). Microbial dry formulations have become ineffective as a result of poor storage conditions (Ramasubramanian et al., 2014). Additional obstacles in generating economically viable microbial pesticides include a slow rate of kill, lack of field persistence due to high UV radiation levels, and poor water solubility of some formulations (Aneja et al., 2016).

## 10. Strategies to address/overcome the ENMs limitations

Although entomopathogenic fungi are being widely utilized as biocontrol agents for insect pest management, they have certain drawbacks, such as varying efficacy, uneven performance in the field, and expensive manufacturing and application costs. There are some instances of approaches that have been suggested to deal with these constraints.

### 10.1. Improved formulation and delivery

Increasing entomopathogenic fungi's formulation and distribution serves as a strategy to boost its potency and uniformity. For instance, Gouli et al. (2021) revealed that incorporating a surfactant into a commercial formulation of the entomopathogenic fungus-*B. bassiana* increased its effectiveness against the tomato leaf miner (*Tuta absolute*).

### 10.2. Enhancing tolerance to environmental stress

A different approach is to make entomopathogenic fungi more tolerant to environmental stresses such as high temperatures, UV radiation, and desiccation. For instance, Hajek et al. (2021) discovered that *M. anisopliae*, an entomopathogenic fungus, may adapt to high temperatures by being exposed to them gradually over numerous generations.

### 10.3. Combining entomopathogens with other biocontrol agents

Combining entomopathogenic fungus with other biocontrol agents, such as parasitoids or predators, may have beneficial consequences. For instance, research by Ekesi et al. (2021) demonstrated that the combination of the parasitic worm, *Fopius arisanus*, and the entomopathogenic fungus *B. bassiana* was more efficient in controlling the invasive fruit fly *Bactrocera dorsalis* than either agent alone.

### 10.4. Developing new strains and species

In addition, another strategy is for developing new entomopathogenic fungal strains or species with enhanced effectiveness, expanded host ranges, or additional desirable characteristics. For instance, research by Fang et al. (2020) encountered a novel strain of *B. bassiana* that was extremely virulent against a predominant pest known as the whitefly, *Bemisia tabaci*. The above strategies indicate how an ongoing investigation is underway to improve entomopathogenic fungi's efficiency as biocontrol agents for effective insect pest management.

## 11. Future perspectives

Entomopathogens serve as promising biocontrol agents with the potential to become a significant component in sustainable pest management. According to a recent study, entomopathogens are preferable to chemical pesticides in several ways, including their ability to target specific pests, a lack of pesticide residue in foodstuffs, and the capacity to lower pesticide resistance (Roy et al., 2006). Genetic engineering and biotechnology advances have opened up new possibilities for developing novel entomopathogens with enhanced efficacy and specificity. For example, researchers are investigating RNA interference to target specific genes in insect pests and developing hybrid entomopathogens that combine the properties of multiple microorganisms (Agrawal et al., 2018). In addition, it can also be integrated with other pest management strategies, such as crop rotation, habitat management, and biological control agents, to enhance their effectiveness. For example, researchers have shown that combining entomopathogens with insect-repellent plants can improve the efficacy of pest management (Winkler et al., 2019). There is limited attention on biopesticides because rural farmers in India have the least agricultural education or training. Outreach activities, such as training and field demonstrations, are essential for promoting the use of entomopathogens as a sustainable pest control method. Outreach activities can provide individuals with a better understanding of the benefits and limitations of using entomopathogens as a pest control method. This knowledge can help individuals decide when and how to use these products. Moreover, proper use of entomopathogens requires specific knowledge and skills. Outreach activities can teach individuals how to apply and manage entomopathogens to maximize their efficacy properly. By promoting entomopathogens, outreach activities can help reduce pesticide use and promote sustainable pest management practices. Several studies have demonstrated the importance of outreach activities for promoting the use of entomopathogens. For instance, a study conducted in Ethiopia found that training workshops for farmers increased knowledge and adoption of entomopathogenic fungi for controlling the coffee berry borer (António et al., 2017). Similarly, a study in India found that field demonstrations and training workshops for farmers improved the adoption of entomopathogenic nematodes for controlling the insect pest root-knot nematode in tomatoes (Kumar et al., 2021). Outreach activities can help increase the adoption of entomopathogens as a pest control method. By educating individuals about the benefits and limitations of entomopathogens and providing them with the necessary knowledge and skills, outreach activities can help them feel more confident using these products. Organizations can offer training workshops, field demonstrations, and educational materials, such as brochures, manuals, and videos, to implement outreach activities for entomopathogen adoption. These activities can target farmers, extension agents, researchers, and other stakeholders involved in pest management. By doing so, we can promote using entomopathogens as a sustainable and effective pest management strategy.

## 12. Conclusion

There has been growing interest in employing biopesticides for protecting crops through sustainable approaches in developing countries. Because the indiscriminate application of synthetic molecules led to resistance development and excessively contaminate the environment. Entomopathogen-based biopesticides are mainly suggested to be incorporated for pest management because of their environmental safety, primarily due to their host specificity. In addition, the cost of formulation and registration of microbial pesticides is much lower than synthetic chemicals. Over the last number of decades, efforts have resulted in the potential application of entomopathogens against various insect pests. However, an investigation is needed to explore many more virulent strains identification, isolation, and formulation preparation to effectively apply them.

Additional work needs to be done to eliminate limitations, including sensitivity to UV rays, desiccation, and low colony counts. Although biopesticides possess several advantages, they are not popularized due to a lack of awareness. It is important to conduct outreach activities, such as training and field demonstrations, to promote the wider acceptance of biopesticide-based formulations among growers. Therefore, insightful information about the potential of microbial insecticides in pest suppression is provided. They have been proven to be an effective component of integrated pest management in managing pest populations below an economic threshold level.

## Author contributions

I and MS conceived and designed the study and prepared the figures/artwork. I, MS, EH, and PR performed the literature search. I wrote the first draft of the manuscript. I, MS, AM, and EK edited the manuscript. I, MS, and EH formatted the reference list as per Journal's style. All authors have approved the final version of the manuscript.
